# Discriminating between Anxious and Non-Anxious Subjects Using the Toronto Hospital Alertness Test

**DOI:** 10.3389/fpsyt.2017.00005

**Published:** 2017-02-02

**Authors:** Colin Shapiro, Lucie Truffaut, Sophie Matharan, Valérie Olivier

**Affiliations:** ^1^Sleep and Alertness Clinic, University Health Network, Toronto, ON, Canada; ^2^Institut de Recherche Internationale Servier, Suresnes, Paris, France

**Keywords:** anxiety, Toronto Hospital Alertness Test, assessment of alertness, self-report scales, psychometric validation

## Abstract

**Background:**

Alertness designates the internal feeling of wakefulness or arousal and is often described to be linked to the level of anxiety. An adequate level of anxiety favoring the alertness needed to deal with a faced specific situation efficiently; too much anxiety can result in failure to process information and respond appropriately. Thus, it would be of interest to verify if different alertness profiles can be observed depending on anxiety level. The Toronto Hospital Alertness Test (THAT) is a test designed to measure alertness. The present survey’s aim is to verify if the THAT allows observing different alertness profile between self-described anxious and non-anxious subjects.

**Methods:**

Subjects >18 years were selected from online databases in three countries (Canada, USA, and UK). All respondents filled in a Hospital Anxiety Depression Scale questionnaire, and only those self-classified as anxious or non-anxious (HAD-A ≥11 or ≤7, respectively) took part to the survey and were asked to complete the THAT.

**Results:**

Among 616 respondents retained in the survey, 414 were self-assessed as anxious and 202 as non-anxious. The mean THAT score for anxious and non-anxious subjects was 21.4 and 38.9, respectively. A receiver operating characteristic (ROC) curve of THAT scores indicated that a threshold score of 30 was required to achieve good sensitivity (86.7%) and specificity (88.6%), with good discriminatory power [an area under the curve (AUC) of 0.938]. As age was determined to be a potential confounder, subjects were age-matched giving a ROC with an AUC of 0.931, with good sensitivity (88.5%) and specificity (89.3%), and the threshold remaining at 30. The internal reliability of THAT in anxious subjects was good (Cronbach alpha = 0.84).

**Limitations:**

No independent verification of anxious or non-anxious status or other eligibility criteria was done.

**Conclusion:**

The alertness profiles of self-defined anxious and non-anxious subjects observed on THAT are different. Based on a subject’s alertness profile, it is possible to discriminate between self-defined anxious and non-anxious, using THAT, with good specificity and sensitivity at a threshold score of 30.

## Introduction

Alertness is defined as the capacity of the mind to respond appropriately to external and internal stimuli. This level of vigilance cannot be defined and limited to an opposite state of daytime sleepiness (1).

It has been shown that hypervigilance and attentional biases to threat are prominent features of the anxious phenotype, and there is growing evidence that they contribute to the development of psychopathology (2). Also, individuals with anxiety disorders exhibit a “vigilance-avoidance” pattern of attention. Finally, stress-induced plasticity within the amygdala is involved in the transition from normal vigilance responses to emotional reactivity, fear overgeneralization, and deficits in fear inhibition resulting in pathological anxiety and conditions, such as panic and depression (3).

To verify if a specific and distinct profile of alertness could be observed in subjects depending on their self-defined level of anxiety would be of interest and could be used in pathological situations to better characterize and follow patients suffering from anxiety disorders.

The Toronto Hospital Alertness Test (THAT) (4) is used to assess alertness and consists of 10 items (measured on a 6-point scale) where individuals self-report their perception of alertness. The items encompass ability to concentrate or focus, i.e., variables that have been associated also with some anxiety disorders. However, the THAT has not yet been specifically used in subjects with anxiety and the alertness profile of anxious or non-anxious subjects has not been described through this test.

An online survey was put in place with the aim of characterizing the alertness profile of subjects who had been self-described [using the Hospital Anxiety Depression Scale (HADS) questionnaire] as either anxious or non-anxious (5) using the THAT to verify if an homogenous profile could be observed in each group and if differences could be highlighted between these two groups of subjects. In two previous studies, healthy subjects were shown to have mean THAT scores of 38 ± 7 [*n* = 12; (6)] and 37 ± 8 [*n* = 1,010; (7)], giving an idea of what might be expected as a typical alertness profile or THAT score for non-anxious individuals. A THAT score, which can reliably profile the alertness of self-defined anxious and non-anxious individuals, would confirm that THAT could be sensible enough to characterize subjects with pathological anxiety. Furthermore, it would also enable to evaluate the effect of treatment in such patients on the variables associated with anxiety that are intrinsic to the THAT.

Here, we evaluated whether it is possible to assign an alertness profile to anxious and non-anxious self-defined subjects using the THAT. Data presented indicate that these both groups of subjects have distinct alertness profiles and allow to identify a THAT threshold score that effectively enable to distinguish between anxious and non-anxious subjects based on their alertness profile. Additionally, some elements of psychometric validation were assessed in self-defined anxious subjects.

## Materials and Methods

### Design

In a period of less than 1 month (25 days), subjects from three different countries, Canada, USA, and UK, completed an online survey concerning their state of anxiousness. The questionnaire took approximately 15 min to complete. Subjects were selected from an online database provided by Vision Critical [Angus Reid Forum, Canada (125,000); Springboard, USA (200,000) and UK (50,000)]. Interviews have been conducted online *via* a national panel in each country (ARF in Canada and Springboard in USA and UK) with an engaging technology on the Sparq platform, which provides a robust and secure environment for authoring questionnaires, choosing sample, deploying studies, and gathering and analyzing data. During the survey process, prospective members were given a clear explanation of what membership of the panel can offer and access to more details should they require these. After completing the profiling questionnaire and agreeing to the terms and conditions, panelists received an email asking them to confirm their desire to join our panel. All answers were anonymized according to the ESOMAR deontology, and participants were informed that the survey results can be published.

The survey was designed to obtain the answers of 400 subjects describing anxious feelings and 200 subjects describing no anxious feelings. All subjects were 18 years of age or over and were low level consumers of alcohol (self-reported). Subjects with severe or chronic disease could not participate unless the progression of the disease had stabilized. Participants completed a HADS questionnaire and responded to a range of questions in order to classify their anxious or non-anxious status. Subjects who had worries—causing them significant distress or impairment in life—present most days for more than 6 months, combined with a HADS-A (anxiety) subscore ≥11 that was greater than or equal to their HADS-D (depression) subscore, were deemed anxious. Participants who were not particularly anxious or excessively nervous about everyday life events, with no worries, and with HADS-A and -D subscores ≤7, were deemed non-anxious. Subjects not fulfilling these thresholds did not take part to the survey. Furthermore, only anxious subjects who had not taken psychotropic treatments in the previous week and non-anxious subjects who had not taken psychotropic treatments in the previous 2 weeks were eligible to participate. Information concerning the subjects’ age, sex, and employment status was also recorded.

### Analysis

The score of each completed THAT, summing 10 variables ranging in score from 0 to 5 each, was calculated. For the first eight variables, 0 represented “not at all” and 5 represented “all the time,” while the last two variables had a reversed scoring system (5 represented “not at all” and 0 represented “all the time”). The distribution of the calculated THAT scores was plotted as a receiver operating characteristic (ROC) curve. The sensitivity and specificity of each score was calculated in order to determine a suitable threshold score that would serve to distinguish between self-defined anxious and non-anxious subjects, according to their alertness profile, using the THAT. The area under the ROC curve (AUC) summarizes the diagnostic utility of the THAT score. In the case of non-informative THAT score (i.e., prediction with the same chance of anxious or non-anxious subjects), the AUC will be 0.5 and will be represented by the diagonal on the ROC graph. The more the AUC is near to 1, the more the THAT score discriminates the self-defined anxious and non-anxious subjects.

Univariate analyses were performed to determine if any of the variables such as age (<35, ≥35 to ≤59, and >59 years), employment status, country, or sex, influenced the alertness profile of the self-defined anxious or non-anxious subjects. This was followed by a stepwise logistic multivariate analysis to determine upon which variable(s) data should be adjusted. Using this stepwise strategy, data were finally adjusted for confounding variables and the threshold score was validated after adjustment. After matching potentially confounding variables at a ratio 2:1, a new population of subjects, with no variation in variables, was identified. The threshold score (without potential confusing bias) of the THAT was assessed using a ROC curve derived from the new sample population.

The internal consistency reliability of the THAT score (estimated using Cronbach’s alpha) and the construct validity of the scale (Spearman’s rank correlation matrix and principal component analysis) was evaluated in self-defined anxious subjects.

## Results

### Subjects Characteristics

A total of 616 subjects participated in the survey, with 414 self-assessed as anxious and 202 as non-anxious (Table 1). Among these subjects, 201 were selected from the Canadian database, 209 from the USA database, and 206 from the UK database. At baseline, 62 and 51% of anxious and non-anxious subjects were females, respectively. A larger proportion of anxious subjects were under 35 years (52%) versus non-anxious (17%) with far fewer anxious subjects over the age of 60 years (5%) than non-anxious (42%). Anxious subjects were more likely to be employed (83%), unlike non-anxious participants where the percentage of employed (56%) and unemployed (44%) was similar (Table 1). The mean HADS-A subscore for anxious and non-anxious patients was 14.4 ± 2.4 and 3.8 ± 2.2, respectively. The mean HADS-D subscores were 9.3 ± 3.3 and 2.8 ± 2.1 for anxious and non-anxious subjects, respectively.

**Table 1:** **Subject characteristics**.

	Self-assessment		All
	Anxious	Non-anxious	
All	414 (67%)	202 (33%)	616 (100%)
Female	257 (62%)	103 (51%)	360 (58%)
Canada	134 (32%)	67 (33%)	201 (33%)
USA	142 (34%)	67 (33%)	209 (34%)
UK	138 (33%)	68 (34%)	206 (33%)
<35 years	214 (52%)	34 (17%)	248 (40%)
35–59 years	178 (43%)	84 (42%)	262 (43%)
≥60 years	22 (5%)	84 (42%)	106 (17%)
Employed	345 (83%)	114 (56%)	459 (75%)
HADS-A	14.4 ± 2.4	3.8 ± 2.2	–
HADS-D	9.3 ± 3.3	2.8 ± 2.1	–

*Values are presented as numbers and percentages (%)*.

*HADS-A, Hospital Anxiety Depression Scale, anxiety subscore; HADS-D, Hospital Anxiety Depression Scale, depression subscore*.

### THAT Score

**Figure 1:**
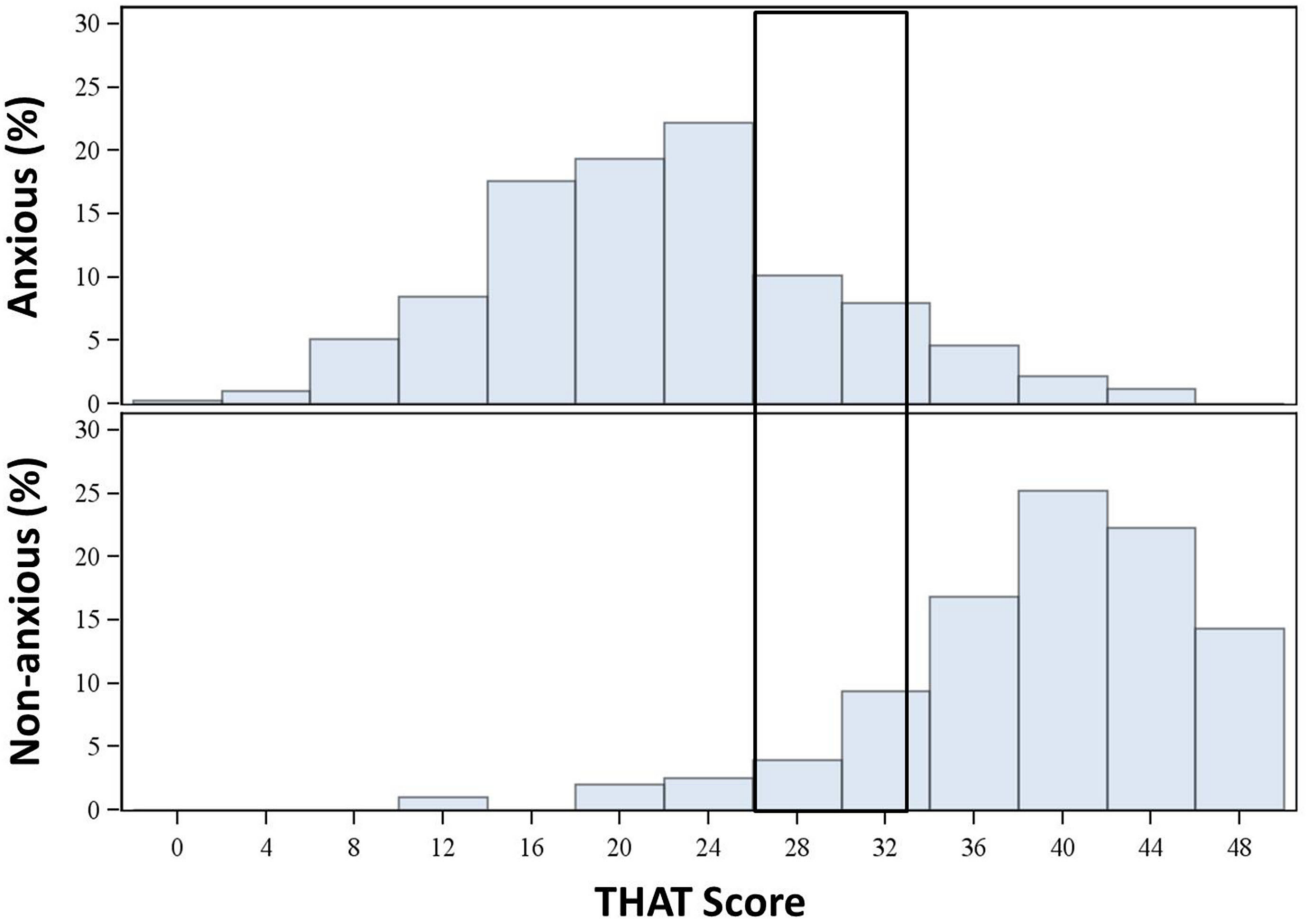
**Distribution of Toronto Hospital Alertness Test (THAT) score in anxious and non-anxious subjects (initial sample)**.

**Figure 2:**
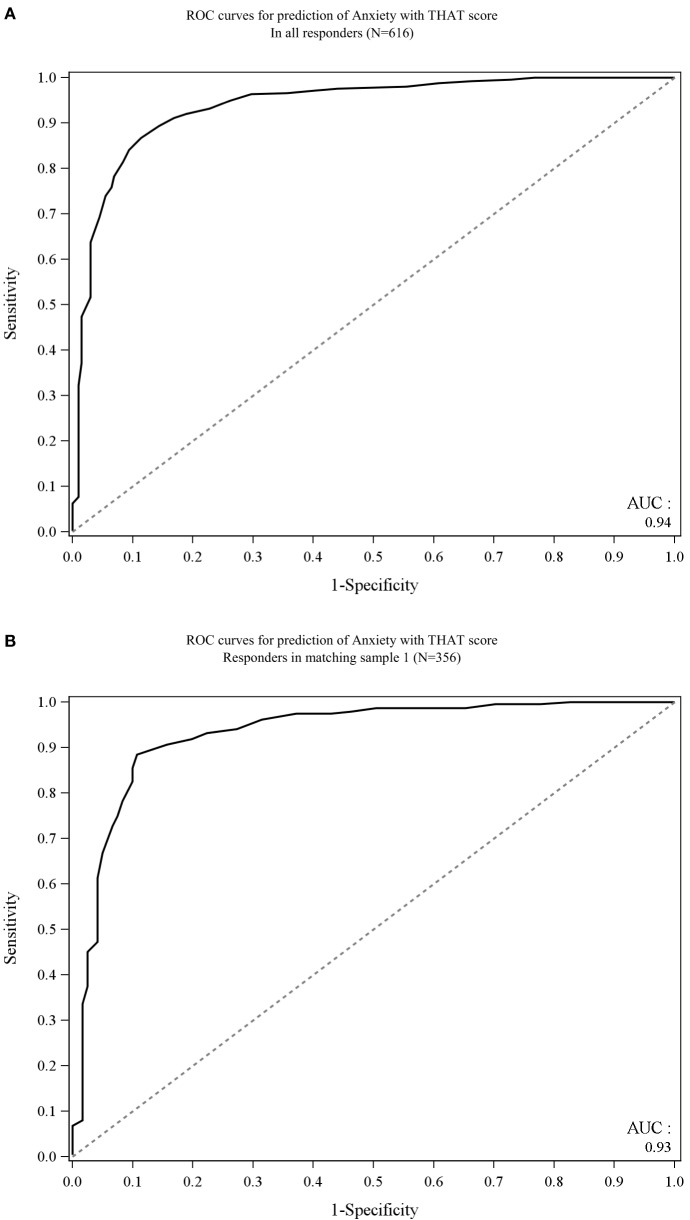
**Receiver operating characteristic (ROC) curve of (A) all anxious and non-anxious subjects (B) age-matched anxious and non-anxious subjects**.

The mean THAT score of anxious subjects was 21.4, while the mean THAT score for non-anxious subjects was 38.9. The THAT scores for anxious and non-anxious subjects in the USA, Canada, and UK were similar (USA, 22.6 and 39.7, respectively; Canada, 21.7 and 39.1, respectively; and UK, 20.1 and 38.0, respectively). The distribution of THAT scores indicated that anxious participants were most likely to have an alertness score ≤28, while non-anxious subjects were more likely to have an alertness score ≥34 (Figure 1). A ROC curve is then used to measure the accuracy of the prediction in identifying anxious subjects using the THAT score. The THAT score had good discriminatory power between anxious and non-anxious subjects, with an area under the curve (AUC) of 0.938. A threshold score of 30 was required to achieve maximized sensitivity and specificity. At this threshold score, the THAT had good sensitivity (86.7%) and specificity (88.6%), with positive- and negative-predictive values of 94.0 and 76.5%, respectively (Figure 2A).

Univariate analyses of all individual characteristic variables indicated that age, employment status, and gender could influence the type of alertness profile that anxious and non-anxious subjects were assigned. Using a stepwise logistic multivariate analysis (entry criterion *p* = 0.3, removal criterion *p* = 0.15), data were finally adjusted on age (<35, ≥35 to ≤59, and >59 years). Therefore, age should be taken into account when determining the THAT threshold score.

A total of 356 subjects were age-matched (using a 2:1 ratio), thereby eliminating the age-bias observed in the initial population, where non-anxious subjects were considerably older than anxious subjects (36.5 ± 12.6 versus 53.1 ± 14.9 years). After subjects were age-matched, the characteristics of anxious and non-anxious subjects were similar except for gender (Table 2).

**Table 2:** **Subject characteristics of age-matched sample and initial sample**.

	Anxious	Non-anxious	All
Unmatched (*n*)	414	202	616
Age (years)			
• Mean ± SD	36.5 ± 12.6	53.1 ± 14.9	42.0 ± 15.5
• Median (Q1; Q3)	33 (26; 46)	56 (42; 64)	40 (29; 55)
Age-matched (*n*)	235	121	356
Age (years)			
• Mean ± SD	43.5 ± 11.6	44.2 ± 11.7	43.7 ± 11.6
• Median (Q1; Q3)	44 (33; 53)	44 (34; 54)	44 (33; 53)
Female	151 (64%)	66 (55%)	217 (61%)
Canada	63 (27%)	25 (21%)	88 (25%)
USA	91 (39%)	56 (46%)	147 (41%)
UK	81 (34%)	40 (33%)	121 (34%)
Employed	190 (81%)	99 (82%)	289 (81%)

*Values are presented as means ± SDs, medians, or numbers and percentages (%)*.

A ROC curve showed that on an age-matched sample the THAT had good powers of discriminating between anxious and non-anxious according to their alertness profile with an AUC = 0.931, while the threshold score maximizing sensitivity and the specificity remained at 30. At this threshold score, THAT had good sensitivity (88.5%) and specificity (89.3%) with positive- and negative-predictive values of 94.1 and 80.0%, respectively (Figure 2B).

### Elements of Psychometric Validation

**Figure 3:**
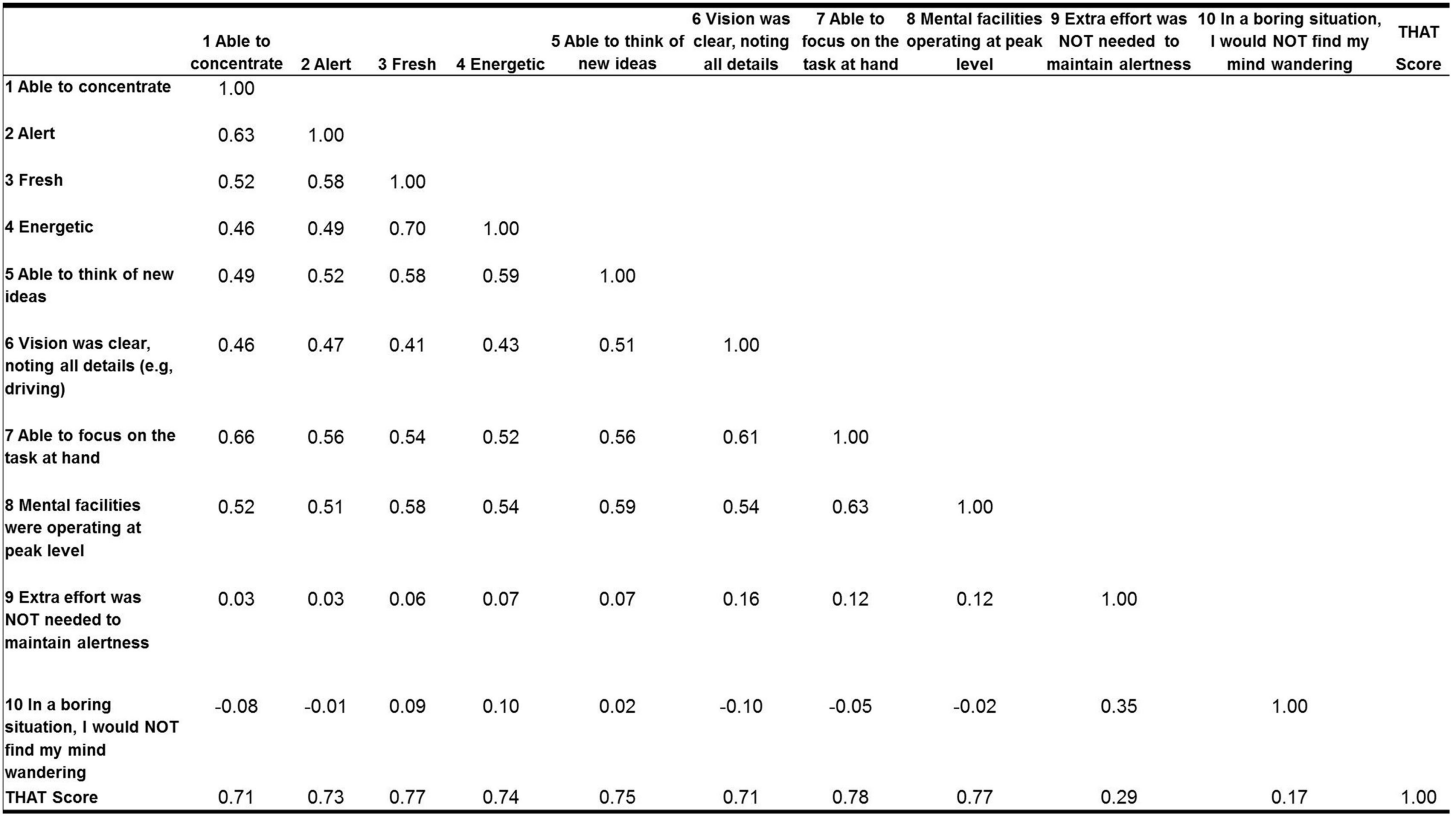
**Spearman rank correlation matrix for the 10 variables in anxious subjects sample**.

A selection of psychometric validation tests of the THAT was performed on anxious subjects. The internal reliability of the THAT in anxious subjects was determined as good (Cronbach alpha = 0.84). The Spearman’s correlation coefficient between items was moderate (between 0.4 and 0.7) and close to 0.7 between items and total score for items 1–8, suggesting good construct validity for those items. However, items 9 and 10 showed no correlation with other items and a weak correlation between those items and total score (0.29 and 0.17, respectively) (Figure 3). Moreover, the first component emerged on the principal component analysis with 48.6% of the variance explained by this first axis. The first component summarizes the information from the items 1 to 8 and the second component describes the information from items 9 to 10.

## Discussion

The data from this survey showed that self-defined anxious and non-anxious subjects have distinct alertness profiles that can be evaluated using the THAT. At a threshold score of 30, the THAT can effectively discriminate between anxious and non-anxious subjects according to their alertness profile. This was observed in both the original and age-matched population samples. The THAT had good sensitivity and specificity in both populations.

The reliability of internal consistency, validated in anxious subjects, was good. The Spearman’s correlation matrix showed a strong correlation for items 1–8 with the total THAT score. However, the correlation for the last two items, 9 and 10, was weak. Furthermore, using a principal component analysis, the last two items were also separated from the first eight items. The inconsistencies of the results concerning the last two items may be due to the phrasing and scoring of the items, with the scoring reversed for items 9 and 10.

Studies by Dean et al. and Ionescu et al. showed that subjects considered as healthy volunteers had mean THAT scores of 38 ± 7 and 37 ± 8, respectively (6, 7). Our survey indicates that subjects with a THAT score >30 can be considered as non-anxious. Although this score is slightly lower than the findings of these previous studies, it is within the same range.

One of the limitations of this study was that all anxious subjects were self-identified *via* the HADS questionnaire only; further research in patients suffering from pathological anxiety in the frame of a clinical trial would be useful to verify if the threshold observed in participants of this survey would be similar in pathological conditions. Additionally, in the present survey, the information is reported by participants with no independent confirmation especially regarding potential psychotropic agent use, presence of severe or unstable chronic disease or alcohol intake, which could also have an impact on alertness level. There were considerably more females than male subjects who were classified as anxious. However, this is in line with other literature, which suggests that more females present with anxiety disorders than males (8, 9).

## Conclusion

This survey shows that self-defined anxious and non-anxious subjects have distinct alertness profiles. The THAT can discriminate between these two groups of subjects according to their alertness profile. A good specificity and sensitivity is observed with THAT at a threshold score of 30.

The results need to be confirmed in a population of patients suffering from anxiety disorder and in specifically designed trials.

## Author Contributions

This work was funded by Servier.

## Conflict of Interest Statement

The authors declare that the research was conducted in the absence of any commercial or financial relationships that could be construed as a potential conflict of interest.
